# Neurotrophins and Neuropathic Pain: Role in Pathobiology

**DOI:** 10.3390/molecules200610657

**Published:** 2015-06-09

**Authors:** Nemat Khan, Maree T. Smith

**Affiliations:** Center for Integrated Preclinical Drug Development, The University of Queensland, St Lucia Campus, Brisbane, Queensland 4072, Australia

**Keywords:** allodynia, BDNF, central sensitisation, NGF, NT-3, NT-4, neurotrophins, neuropathic pain, p75NTR, neurotrophic tyrosine kinase (Trk) receptor

## Abstract

Neurotrophins (NTs) belong to a family of trophic factors that regulate the survival, growth and programmed cell death of neurons. In mammals, there are four structurally and functionally related NT proteins, *viz*. nerve growth factor (NGF), brain-derived neurotrophic factor (BDNF), neurotrophin 3 and neurotrophin 4. Most research on NTs to date has focussed on the effects of NGF and BDNF signalling via their respective cognate high affinity neurotrophic tyrosine kinase viz TrkA and TrkB receptors. Apart from the key physiologic roles of NGF and BDNF in peripheral and central nervous system function, NGF and BDNF signalling via TrkA and TrkB receptors respectively have been implicated in mechanisms underpinning neuropathic pain. Additionally, NGF and BDNF signalling via the low-affinity pan neurotrophin receptor at 75 kDa (p75NTR) may also contribute to the pathobiology of neuropathic pain. In this review, we critically assess the role of neurotrophins signalling via their cognate high affinity receptors as well as the low affinity p75NTR in the pathophysiology of peripheral neuropathic and central neuropathic pain. We also identify knowledge gaps to guide future research aimed at generating novel insight on how to optimally modulate NT signalling for discovery of novel therapeutics to improve neuropathic pain relief.

## 1. Introduction

**Figure 1:**
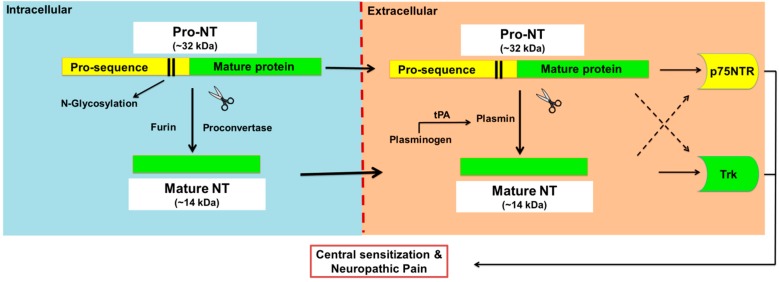
Proteolytic cleavage of pro-neurotrophins to mature neurotrophins. Neurotrophins (NTs) are synthesised as glycosylated precursors of ~32 kDa, called pro-neurotrophins, pro-NTs. These precursor molecules are cleaved intracellularly by furin or proconvertase, and extracellularly by the action of plasmin to generate mature NTs. Plasmin is a serine protease enzyme that is formed from plasminogen by several enzymes including tissue plasminogen activator (tPA) and urokinase. Intact pro-NTs may also be released extracellularly and evoke direct biological activities that in general oppose that of the mature NT counterpart although there are exceptions. For example, pro-NTs may induce apoptosis whereas mature NTs are mainly associated with neuronal survival. However, both pro- and mature-NTs appear to mediate central sensitisation and neuropathic pain.

Neurotrophins (NTs) are a family of four structurally and functionally related proteins that regulate the growth, maintenance and apoptosis of neurons in the developing nervous system as well as injured neurons [1,2,3]. In mammals, there are four NTs, *viz*. nerve growth factor (NGF), brain-derived neurotrophic factor (BDNF), neurotrophin 3 (NT-3) and neurotrophin 4 with the latter also known as neurotrophin-5 (NT-4 or NT4/5) [1,2]. Initially, NTs are synthesised as precursor molecules called proneurotrophins (pro-NTs) at ~30–34 kDa, that undergo proteolytic cleavage in the endoplasmic reticulum and Golgi apparatus to produce *C*-terminally mature neurotrophins at ~13 kDa [4] (Figure 1). 

**Figure 2:**
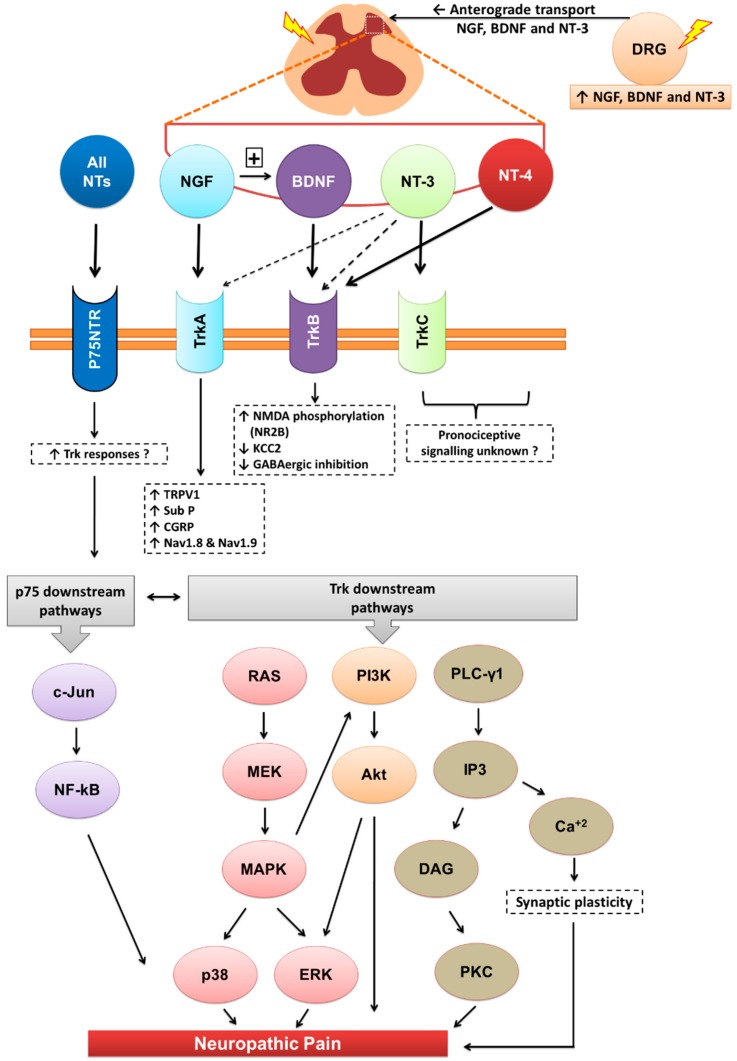
Downstream signalling of neurotrophins and their receptors. Akt, Akt kinase; Ca+2, calcium ions; CGRP, calcitonin gene related peptide; DAG, diacylglycerol; DRG, dorsal root ganglia; ERK, extracellular-signal-regulated kinase; GABA, γ-Aminobutyric acid; IP3, inositol tris-phosphate; KCC2, potassium chloride cotransporter 2; MAPK, mitogen-activated protein kinases; MEK, mitogen-activated protein kinase/ERK kinase; Nav, sodium-ion voltage-gated channel; NMDA, *N*-methyl-d-aspartate; NT, neurotrophin; p75NTR, pan neurotrophin receptor at 75kDa; PI3K, phosphatidylinositol 3-kinase; PLC-γ1, phospholipase C-gamma-1; Ras, small GTP-binding protein; Sub-P, substance-P; Trk, tyrosine kinase receptor; TRPV1, transient receptor potential cation channel subfamily V member 1.

The biological effects of mature NTs are mediated via two major receptor types, viz neurotrophic tyrosine kinase (Trks) receptors and the pan neurotrophin receptor at 75 kDa (p75NTR) (Patapoutian and Reichardt, 2001). Each mature NT binds with high affinity to a specific Trk receptor. Specifically, NGF binds with high affinity to TrkA, BDNF and NT-4 bind with high affinity to TrkB, and NT-3 binds with high affinity to TrkC [5]. Apart from activation of TrkC, NT-3 also activates TrkA and TrkB, albeit with lower affinities [6] and all four mature NTs bind with similar affinity to the p75NTR [5] (Figure 2). By comparison, the biological actions of pro-NTs are relatively poorly understood [7]. Intact pro-NTs are high affinity ligands at both the p75NTR and the structurally distinct co-receptor, sortilin, in contrast to mature NTs [4,8]. Additionally, pro-NGF and pro-BDNF bind to TrkA [9] and TrkB [10] respectively, albeit with lower affinity than to the p75NTR [10,11]. This complexity of endogenous NT signalling has made investigation of potential pathobiological role(s) of pro-NTs particularly challenging. 

The neurotrophins (NTs) particularly NGF, BDNF and NT-3 are synthesised mainly in the dorsal root ganglia (DRG) with anterograde transported into the dorsal horn of the spinal cord. Apart from their roles in various physiological functions, they modulate central sensitisation in the spinal cord that underpins maintenance of neuropathic pain. NTs bind with high affinity to their respective cognate tryosine receptor kinase (Trk) receptors, namely, NGF with TrkA, BDNF and NT4 with TrkB, and NT3 with TrkC. NT3 may also activate TrkA and TrkB, but with lower affinity. All NTs including their precursors (pro-NTs) may bind to the p75NTR and this is the high affinity receptor for pro-NTs. NGF-TrkA signalling is mostly associated with upregulation of TRPV1 expression and insertion into the nociceptor membrane, substance-P, CGRP and the sodium channels, Na_v_1.8 and Na_v_1.9. BDNF-TrkB signalling results in NMDA receptor activation and downregulation of the potassium chloride cotransporter 2 (KCC2) and/or GABAergic inhibitory signalling mechanisms. NT-3 reportedly evokes antinociception whereas NT-4 appears to be predominantly involved in proprioception. Following Trk receptor activation, the downstream signalling mechanisms include the Ras-MAPK, PI3K and PLC-γ1 pathways whereas following activation of the p75NTR, downstream signalling associated with neuropathic pain appears to involve the c-Jun-NF-kB pathway. Additionally, activation of p75NTR may potentiate Trk-mediated signalling in neuropathic pain. 

## 2. Neuropathic Pain

Neuropathic pain is defined by the International Association for the Study of Pain (IASP) as pain that arises as a direct consequence of a lesion or disease affecting the somatosensory system [12]. Neuropathic pain is often poorly alleviated by first- and second-line medications recommended by the Neuropathic Pain Special Interest Group of the IASP due to lack of efficacy and/or dose-limiting side-effects [13,14]. Hence, there is an urgent need to develop novel mechanism-based therapeutic agents that are highly efficacious and well tolerated to improve relief of neuropathic pain [13,14]. 

## 3. Neurotrophins and Neuropathic Pain

NTs contribute to the pathogenesis of neuropathic pain as they have key roles in the complex mechanisms that underpin peripheral and central sensitisation [15,16,17]. However, knowledge on the specific contribution of individual neurotrophins signalling via a particular Trk receptor and/or the p75NTR in the pathobiology of neuropathic pain is required to identify the optimal targets for use in discovery programs aimed at producing novel analgesics for improving neuropathic pain relief.

Adding to the complexity, NGF and BDNF in particular, reportedly evoke both pronociceptive and antinociceptive effects, with the latter being produced by lower doses particularly when administered directly into central nervous system (CNS) [18,19] (Table 1).

## 4. Nerve Growth Factor (NGF)

NGF was the first neurotrophin identified as having a key role in the survival and function of sensory and sympathetic neurons in the peripheral nervous system (PNS), as well as basal forebrain cholinergic neurons in the CNS [20,21].

**Table 1:** Expression of neurotrophins and their nociceptive role in rodent models of neuropathic pain.

NTs	Rodent Models of Neuropathic Pain					
	Tissue	Nerve Ligation/Axotomy(e.g., CCI, SNL)	CIPN	DPN	EAE (MS)	SCI
**NGF**	**DRG**	↑ [22,23]	↑ [24] or ↓ [25]	↑ [26] or ↓ [27]	NS	NS
	**SC**	↑ [23]	↑ [24,28]	↓ [27]	↓ [29,30] (Correlation with pain not investigated)	Not changed [31] or ↑ [32] (Indirect evidence)
	**Pronociception**					
	● Micro-injected NGF (50 µg) via a catheter into an L5 DRG of un-injured rodents, induced ipsilateral persistent mechanical allodynia [33]					
	● Intrathecal (i.t.) infusion of NGF (12 µg/day) for 9 days induced thermal hyperalgesia in rats [34]					
	● Intraplantar (i.pl.) injection of NGF (0.3–5 µg) into a hindpaw produced dose-dependent mechanical allodynia and thermal hyperalgesia that persisted for ≥1 week and 24–48 h respectively [35]. Both mechanical allodynia and thermal hyperalgesia were partially attenuated by a TRPV1 antagonist [35] suggesting that NGF-mediated hyperalgesia is driven at least in part by increased TRPV1 expression [35,36]					
	● NGF (1.0 mg/kg s.c.) significantly increased Sub-P and CGRP expression levels in the dorsal horn of the spinal cord [37] thereby producing neuropathic pain [38]					
	● Overexpression of NGF may induce dysregulation of DRG Na+ channel expression, particularly Nav 1.8 (SNS/PN3), Nav 1.9 (NaN), Nav 1.6, Nav 2.1 (NaG) as well as various Na^+^ channel subunits including α-I, α-II, β-I and β-II in sensory neurons leading to neuropathic pain symptoms [39,40,41,42 ]					
	**Antinociception**					
	● I.t. infusion of NGF (125 ng/µL/h) for 7 days reversed mechanical allodynia and thermal hyperalgesia in the hindpaws of CCI-rats. The analgesic effects of NGF were correlated with neuroprotection and decreased astrocytosis [43]					
**BDNF**	**DRG**	↑ [44]	NS	↑ [45]	↑ [46] (Not investigated in relation with pain)	NS
	**SC**	↑ [44]	↑ [28]	NS	↑ [47]	↑ [48]
	**Pronociception**					
	● Microinjection of BDNF (27–270 pg) into the midbrain facilitated nociception dependent on phosphorylation of NMDA receptors [49]					
	● Micro-injection of BDNF (50 μg) into an L5 DRG of control (non-injured) rodents induced persistent mechanical allodynia in the hindpaws [33]					
	● I.pl. injection of BDNF (200 ng) into rodent hindpaws produced transient thermal hyperalgesia and was significantly less potent (*p* < 0.05) than a similar dose of NGF [36]					
	● In rodent models of peripheral neuropathic pain, upregulated BDNF induced phosphorylation of the NR2B subunit of the NMDA receptor [50] that was accompanied by downregulation of Kv channels [45] as well as expression levels of KCC2 in lamina-I of the spinal cord, thereby disrupting GABAergic inhibition [51]					
	● Migration of inflammatory cells into the spinal cord may contribute to upregulation of BDNF in rodent models of peripheral neuropathic pain (e.g., DPN) [52] or CNP (e.g., MS-neuropathic pain) [47]					
	**Antinociception**					
	● BDNF-infusion (12 µg/day) into the midbrain for 1–11 days evoked antinociception in the tail flick test in rats [18]. The proposed mechanism was via activation of descending opiodergic and serotonergic inhibitory signalling [18]					
**NT-4**	**DRG**	NS	NS			
	**SC**	↓ [53] (Short-term) or Unchanged [54]				
	**Lack of a role in neuropathic pain**					
	● NT-4 appears to have no effect on activity-dependent synaptic plasticity or neuropathic pain [55,56].					
	● In one study, transient thermal hyperalgesia was observed in rats followed by i.pl. injection of NT-4 (200 ng) however it was worn off by 24 h [36]					
	● Although there was a significant decrease in NT-4 expression levels in rodent models of neuropathic pain, e.g., DPN (at sciatic nerve) [57] and EAE (at brain) [58], it’s possible role in the pathogenesis of neuropathic pain remains to be investigated.					
**NT-3**	**DRG**	↑ [22]	↓ [59] (Indirect evidence)	↓ [60]	NS	NS
	**SC**	↑ [61]	NS	NS	↑ [62] (Not investigated in relation with pain)	↑ [63] or ↓ [31] (Not investigated in relation with pain)
	**Pronociception**					
	● Micro-injection of NT-3 (50 µg) into the L5 DRGs of control non-injured rodents produced transient mechanical allodynia in the hindpaws [33]					
	● I.t infusion of NT-3 at 200 ng/day for 20-days produced pronounced but delayed mechanical allodynia in the hindpaws of non-injured rats at days 10–20 after dose initiation [64]					
	**Antinociception**					
	● NT-3 infusion (12 µg/day) into the midbrain for 1–11 days showed delayed but stable antinociception in the tail-flick test in rats [18]					
	● I.t. administration of NT-3 (600 ng/μL/h) for 7 days suppressed the over-expression of TRPV1 channels, p38 MAPK and Na+ channels (Nav 1.8 and Nav 1.9) in the ipsilateral DRGs of CCI-rats [65,66]					
	● Down-regulation of Kv channel gene expression in DRG neurons following sciatic nerve transection was reversed by *ex vivo* incubation of DRGs collected from nerve-injured rats, with NT-3 (100 ng/mL) [67]					
	● Acute i.p. injection of NT-3 (10–20 mg/kg) evoked transient mechanical but not thermal hypoalgesia in the hindpaws of rats that appeared to be underpinned by inhibition of SP release in the spinal cord [68]					
	● NT-3 (200 ng) injected locally into rodent hindpaws did not produce hyperalgesia in contrast to that evoked by either NGF or BDNF [36]					

BDNF, brain derived neurotrophic factor; CCI, chronic-constriction nerve injury; CGRP, calcitonin gene related peptide; CIPN, chemotherapy-induced peripheral neuropathy; DPN, diabetic peripheral neuropathy; DRG, dorsal root ganglia; EAE, experimental autoimmune encephalomyelitis; GABA, γ-Aminobutyric acid; h, h; I.pl, intraplantar; i.t, intrathecal; KCC2, potassium-chloride co-transporter; K_v,_ potassium voltage-gated channel; MAPK, mitogen-activated protein kinases; MS, multiple sclerosis; mg, milligram; mL, millilitre; ng, nanogram; Na^+^, sodium ion; Na_v,_ sodium voltage-gated channel; NGF, nerve growth factor; NMDA, *N*-methyl-d-aspartate receptor; NS, not studied; NT, neurotrophins; s.c, subcutaneous; SC, spinal cord; SCI, spinal cord injury; SNL, spinal nerve ligation; Sub-P, substance-P; TRPV1, transient receptor potential cation channel subfamily V member 1; µg, microgram. µL, microliter; ↑, upregulation; ↓ downregulation.

## 5. Pronociceptive Effects of NGF in Rodent Models 

Following its local or systemic administration in rodents and humans, NGF produced non-inflammatory and long-lasting thermal and mechanical hyperalgesia, with these two pain behaviours mediated by distinct mechanisms [35,69,70,71]. Specifically, NGF-induced thermal hyperalgesia appeared to be underpinned by sensitisation of peripheral nociceptors whereas NGF-induced mechanical hyperalgesia was predominantly mediated by complex signalling in the spinal cord [69,72]. Cross-talk between sensory and adrenergic inputs from sympathetic neurons due to abnormal sprouting of axons from sensory and sympathetic DRG neurons to form baskets around large diameter neurons has also been implicated in the pathogenesis of peripheral neuropathic pain in rodents [22,73,74,75,76].

NGF has been shown to dynamically regulate the synthesis of multiple neurotransmitters and neuropeptides in sensory and sympathetic neurons [20]. These included norepinephrine (noradrenaline) in sympathetic neurons via selective induction of the key enzyme, tyrosine hydroxylase (TH) [77] and the pronociceptive neuropeptides, substance P (SP) and calcitonin gene-related peptide (CGRP) in sensory neurons in the dorsal root ganglia (DRGs) and spinal cord [78]. 

In rats, observations that localised hyperalgesia induced by intraplantar (i.pl.) NGF was attenuated partially by oral pre-treatment with a transient receptor potential vanilloid 1 (TRPV1) antagonist, implicated a role for TRPV1 at least in part, in mediating this pain behaviour [35]. These findings supported earlier work whereby upregulated NGF-TrkA signalling phosphorylated (activated) TRPV1 and promoted its insertion into the nociceptor cell membrane that in turn induced long-term hyperexcitability of primary afferent sensory nerve fibres [79]. In other work, a critical role for NGF-p75NTR signalling has been implicated in sensory nerve fibre hyperexitability and development of mechanical hyperalgesia induced by i.pl. NGF injection [80,81].

In primary sensory neurons, the cannabinoid CB1 receptor is co-localised with substance P, CGRP [82,83] and TRPV1 [83,84], expression levels of which are markedly increased in the cell membrane by retrograde transport of NGF to the cell bodies in the DRGs. In cultured neurons, CB1 receptor activation by cannabinoids attenuated NGF-induced TRPV1 sensitisation [85]. Hence, investigation of the extent to which cannabinoids attenuate NGF-induced TRPV1 upregulation and sensitisation of primary sensory neurons in rodent models of neuropathic pain is warranted. However, the widespread distribution of CB1 receptors in the CNS results in a plethora of cannabinoid-induced side effects including sedation, dependence, motor and cognitive impairments [86,87] that are impediments to the clinical exploitation of cannabinoid inhibition of the pronociceptive effects of NGF to alleviate chronic pain. Interestingly, endocannabinoid expression levels are significantly upregulated both spinally and supraspinally in rodent models of neuropathic pain [86,88,89]. Hence, a strategy for treatment of neuropathic pain worthy of future investigation is administration of a cannabinoid CB2 receptor agonist to down-regulate TRPV1 expression levels in primary sensory neurons and inhibit nociceptive input into the dorsal horn of the spinal cord [87]. This strategy has appeal as CB2 receptor expression is predominantly in peripheral components of the somatosensory system thereby avoiding CNS side effects [87].

## 6. Antinociceptive Effects of NGF in Rodent Models

Apart from variable reports on expression levels of NGF in the pathobiology of peripheral neuropathic pain conditions, the effect of intrathecal (i.t.) NGF on nociception in rodents is also controversial. For example, in one study thermal hyperalgesia was induced by i.t. NGF administration for 9-days at 12 µg/day [34] whereas in other work, chronic i.t. NGF infusion for 7-days at 125 ng/h induced pain relief in a rat model of mechanically-induced peripheral neuropathic pain [43]. The latter effected appeared to involve reduced reactive gliosis and restoration of homeostatic conditions in the spinal cord [43]. 

## 7. NGF: Role in Peripheral Neuropathic Pain 

### 7.1. NGF in Rodent Models of Peripheral Nerve Ligation 

In rodent models of peripheral neuropathic pain induced by peripheral nerve ligation, the pronociceptive action of NGF was reversed by an NGF antagonist [90,91,92]. In parallel with these reports, systemic administration of an antibody against the high affinity NGF receptor, TrkA, produced long lasting anti-allodynia (pain relief) in a mouse model of peripheral neuropathic pain induced by loose ligatures tied around a single sciatic nerve (CCI-mice) [93]. 

### 7.2. Rodent Models of PDN and CIPN: Controversial Reports of NGF Expression 

Two to three decade ago, there were multiple reports of reduced levels of NGF and its regulated neuropeptides (CGRP and SP) in DRG sensory neurons and the spinal dorsal horn in rodent models of painful diabetic neuropathy (PDN) and chemotherapy induced peripheral neuropathy (CIPN), that are peripheral neuropathic pain models [25,28,94,95,96]. In PDN, peripheral neurodegeneration and impaired catecholaminergic neurotransmission were linked to decreased NGF expression [97]. Additionally, incubation of cultured adult rat DRG neurons with chemotherapy agents decreased neurite outgrowth and this was reversed by NGF treatment, suggesting that NGF treatment may be beneficial in CIPN [98]. In support of this notion, there was a correlation between CIPN severity in humans and the decrease in circulating NGF levels [99]. In rodent models of CIPN, NGF treatment reversed the behavioural and biochemical manifestations of pain [25,28,100]. In other work, NGF treatment reversed the downregulated expression of CGRP and SP in the lumbar DRGs of rodent models of PDN and CIPN and alleviated sensory deficits in these animals [25,95,96,100]. 

Conversely, more recent reports indicate that NGF expression is increased in rodent models of neuropathic pain or pain with a neuropathic component [24,101,102,103,104]. The upregulated levels of NGF appear to be located in Schwann cells and satellite glial cells in close proximity to injured primary sensory neurons [105,106]. NGF may also be released by invading mast cells, eosinophils, lymphocytes and macrophages at the site of peripheral nerve injury [22,107,108,109]. Following internalisation of the NGF-TrkA complex, its retrograde transport to the DRGs induced phenotypic changes in the peripheral and central terminals of sensory nerve fibres [38,110]. This in turn increased synthesis and release of pronociceptive mediators including SP, CGRP [111] and BDNF from TrkA-positive primary afferents [112]. The net effect was development of mechanical and/or thermal hyperalgesia in the hindpaws [112].

## 8. NGF: Role in Central Neuropathic Pain (CNP)

### 8.1. NGF in Spinal Cord Injury Induced Neuropathic Pain Rodent Model

Evidence supporting a role for NGF in the pathobiology of various CNP conditions is scarce. Following hemisection of the spinal cord in rats, mechanical and thermal allodynia were induced in the hindpaws [32,113]. These changes were accompanied by elevated levels of CGRP bilaterally in the spinal dorsal horn and by sprouting of CGRP-positive fine sensory afferents from laminae I-II into deeper laminae (III-IV) in the spinal cord [32,113]. A pathobiologic role for NGF in these neuroplastic changes in the spinal cord is supported by observations that i.t. treatment with an anti-NGF antibody for 2-weeks after spinal cord hemisection, prevented CGRP upregulation as well as the sprouting of CGRP-containing primary afferent C- and Aδ-fibres in the spinal cord [32,114]. 

### 8.2. NGF in Multiple Sclerosis-Associated Neuropathic Pain Mouse Model 

Multiple sclerosis (MS) is an inflammatory demyelinating disease of the CNS where debilitating CNP may occur early in the disease course [115]. In patients with MS, upregulated synthesis of NGF by oligodendrocytes, astrocytes, microglia as well as by infiltrating T-lymphocytes and macrophages [116,117] has been proposed to have a role in the promotion of myelin repair as well as attenuation of neuroinflammation [117,118]. 

However, spinal dorsal horn expression levels of the pronociceptive peptides, CGRP and galanin, in an EAE mouse model of MS-associated neuropathic pain did not differ significantly from the corresponding levels in sham-mice administered Freund’s Complete Adjuvant (FCA) only [119]. Hence, these findings appeared to discount a role for upregulated NGF in the pathogenesis of MS-induced CNP [119]. However, the sham-mice had received FCA as adjuvant in the EAE-immunisation protocol, which itself has been shown to induce robust neuroinflammation [120,121] and increase CGRP expression [122]. Hence, an FCA-induced increase in CGRP expression levels in the spinal dorsal horn of sham-mice may have masked a similar effect by MOG_35–55_ in the EAE-mice. Hence, re-examination of this issue using an EAE immunisation protocol that uses an adjuvant (e.g., Quil A) that does not itself produce neuroinflammation [47,123] is warranted to assess the extent to which NGF has a role in the pathobiology of MS-associated neuropathic pain. 

## 9. BDNF

BDNF is another neurotrophic factor implicated in the regulation of pronociceptive signalling in inflammatory and neuropathic pain conditions [124,125]. However, the pathobiologic role of BDNF-TrkB signalling in peripheral neuropathic pain [126], appears to be confined primarily to spinal and supraspinal sites [49,127,128]. This is in contrast to the predominant effects of NGF in the peripheral nervous system (PNS) [102]. Additionally, BDNF-p75NTR signalling, like NGF-p75NTR signalling, may also induce the downstream sphingomyelin-signalling cascade resulting in hyperexitability of small diameter sensory neurons [129]. 

## 10. Pronociceptive Effects of BDNF in Rodent Models

Increased BDNF expression in the spinal dorsal horn of rats with spinal nerve injury-induced neuropathic pain peaked at 24–48 h and was highly correlated with the onset of neuropathic pain behaviour [50,130]. Conversely, BDNF-mediated phosphorylation of the NR2B subunits of *N*-methyl-d-aspartate (NMDA) receptors was associated with the maintenance phase of neuropathic pain [50,130]. As the selective NR2B antagonist, Ro25-6981, markedly attenuated neuropathic pain in similarly nerve-injured rodents, a key role for NR2B-containing NMDA receptors in the maintenance of neuropathic pain, was affirmed [50]. 

The rostral ventromedial medulla (RVM) and the periaqueductal gray (PAG) matter are brain regions thought to have critical roles in descending facilitation of pronociceptive signalling in neuropathic pain [131]. In support of this notion, microinjection of BDNF (10–100 fmol) into the RVM facilitated nociception that was dependent on NMDA receptor activation [49]. Activity-dependent BDNF release from spinal neurons as well as supraspinal neurons in the hippocampus, PAG and RVM [49,132], was shown to underpin BDNF-induced descending facilitation [49,132]. Additionally, in a peripheral nerve ligation rat model of neuropathic pain, increased BDNF-TrkB signalling induced activation of phosphorylated-p44/p42 mitogen activated protein kinase (also called phospho-ERK, pERK) in the RVM that was correlated significantly with development of mechanical allodynia, a defining symptom of neuropathic pain [128]. 

## 11. Antinociceptive Effects of BDNF in Rodent Models

Contrary to the pronociceptive effects of BDNF in the RVM and PAG brain regions, antinociception was evoked by BDNF infusion (12 µg/day) into the midbrain of non-injured rats that appeared to be mediated by opioidergic signalling as well as increased serotoninergic activity both supraspinally and spinally [18]. Additionally, supraspinal release of BDNF particularly in the parabrachio-amygdala signalling pathway appears to have a key role in the mediation of morphine-induced analgesia [133]. In other work, neuropathic pain behaviour was abolished by recombinant adeno-associated viral (rAAV)-mediated BDNF over-expression in the spinal cord [134] or transplantation of genetically engineered BDNF-secreting neurons into the spinal cord of CCI-rats [135]. Hence, the net effects of sensory neuron derived BDNF on nociception in the dorsal horn are complex, in a manner similar to NGF [18,134,135]. 

Apart from pharmacological treatment of neuropathic pain, a considerable body of research in rodents shows that non-pharmacological interventions, such as electro-acupuncture (EA) at 2–10 Hz, alleviate neuropathic pain [136]. The underpinning analgesic mechanisms are complex and include attenuation of pronociceptive signalling and/or upregulation of antinociceptive signalling at multiple levels of the somatosensory system [136]. Of interest herein, EA-induced relief of neuropathic pain in rodents was accompanied by upregulated CNS expression of NTs particularly BDNF and NT-3 [137,138]. In neuropathic rats, EA-evoked pain relief was also accompanied by increased endogenous opioid peptide expression at peripheral nerve terminals, the spinal cord and brain, as well as enhanced descending noradrenergic and serotonergic neurotransmission [136,137,138,139,140]. This in turn inhibited NMDA receptor activation and glial cell activation in the spinal cord, with the net effect being decreased proinflammatory cytokine expression levels in the spinal dorsal horn [136,137,138,139,140].

## 12. BDNF: Role in Peripheral Neuropathic Pain 

### 12.1. BDNF in Rodent Models of Peripheral Nerve Ligation 

Peripheral nerve injury in rodents induced an upregulation in the synthesis and release of BDNF in small, medium and/or large diameter DRG neurons and in the superficial laminae of the spinal dorsal horn as well as in the gracile nuclei [44,124]. In more recent work, peripheral nerve injury on post-natal day 10 in infant rats did not induce neuropathic pain behaviour and this was correlated with a predominantly anti-inflammatory cytokine (IL-4 and IL-10) profile in the spinal dorsal horn of these animals [141]. However, once these same animals reached adolescence from post-natal day 21 onwards, the neuropathic pain behavioural phenotype became evident and this was accompanied by a switch from an antinociceptive to a pronociceptive cytokine/growth factor profile in the spinal dorsal horn [141]. Specifically, there was marked upregulation of spinal dorsal horn expression levels of BDNF and tumor necrosis factor alpha as well as a marked down-regulation of spinal dorsal horn expression levels of the anti-inflammatory cytokines, IL-4 and IL-10 [141]. These findings collectively suggest that although peripheral nerve injury in infancy may not appear to produce neuropathic pain due to powerful inhibition by anti-inflammatory cytokines in the spinal dorsal horn, there is a risk that it may emerge for no apparent reason in adolescence following switch of the neurochemical profile in the spinal dorsal horn from antinociceptive to pronociceptive [141]. 

Expression of TrkB, the high affinity BDNF receptor, was also upregulated in the lumbar DRGs and spinal dorsal horn [142,143]. In rodents with peripheral nerve injury, BDNF, like NGF, promoted sympathetic sprouting in the lumbar DRGs [74,144] and this was prevented by i.t. infusion of BDNF antiserum [145]. Affirming a role for augmented spinal BDNF-TrkB signalling in the pathobiology of neuropathic pain were observations that repeated i.t. administration of anti-BDNF or a BDNF-sequestering TrkB-Fc chimera protein, abolished neuropathic pain behaviours [55,146].

In rat neuropathic pain models, upregulated BDNF expression by an NGF-dependent mechanism in DRG neurons resulted in anterograde transport of BDNF by axonal mechanisms into the spinal dorsal horn [130,147,148], such that its release was restricted to TrkA-positive neurons in both lumbar DRGs and the spinal dorsal horn [112,147,149].

### 12.2. BDNF in Rodent Models of PDN 

Investigation of neurotrophins other than NGF in the pathogenesis of PDN is in its infancy [150]. In diabetic rats, increased BDNF expression in the lumbar DRGs [151] was linked with decreased expression of voltage-gated potassium (K_v_) channels in the lumbar DRGs and primary sensory neuron hyperexcitability [45]. This decreased K_v_ channel expression in DRG neurons from diabetic rats was reversed by treatment with anti-BDNF antibody for 2–4 h [45]. Mimicking these observations in diabetic rats, mRNA levels for NGF, BDNF and NT-3 and their receptors were increased in peripheral nerves with axonal pathologies in patients with PDN [52]. The upregulated BDNF and NT-3 mRNA levels in the diseased segments of peripheral nerves was positively correlated with the extent of nerve invasion by T-cells and macrophages rather than axonal pathology or demyelination [52]. Thus neuroinflammation appears to be a key pathobiologic event in PDN.

### 12.3. BDNF in Rodent Models of CIPN 

A role for BDNF in the pathogenesis of CIPN in rodent models or in patients is scant. In a single study using a rat model of CIPN, BDNF expression in the spinal dorsal horn was upregulated compared with the corresponding level in sham-animals [28]. Clearly, additional research is warranted. 

## 13. BDNF: Role in Central Neuropathic Pain 

### 13.1. BDNF in Spinal Cord Injury Induced Neuropathic Pain Rodent Model 

In a rat model of spinal cord injury-induced CNP, a pathobiologic role for upregulated BDNF-TrkB signalling in the spinal cord is well established [48]. In particular, the truncated TrkB receptor isoform expressed by both neuronal and non-neuronal cells is implicated in the pathogenesis of SCI-induced CNP as expression levels were consistently increased in both white and grey matter in spinal cord tissue [48,152]. 

### 13.2. BDNF in Multiple Sclerosis-Associated Neuropathic Pain Mouse Model

In MS, upregulated BDNF expression in the CNS is due to infiltration of BDNF-containing T-cells and upregulated synthesis by microglia and reactive astrocytes rather than spinal cord neurons [153,154]. In recent work from our laboratory that used our novel optimised relapsing-remitting EAE (RR-EAE) mouse model of MS-neuropathic pain [123], mature BDNF was found to be down-regulated whereas its precursor, pro-BDNF, was upregulated in the lumbar spinal cord of RR-EAE mice exhibiting pain behaviour [47]. Thus the possibility that pro-BDNF has a pronociceptive role in the pathobiology of MS-associated neuropathic pain, requires future investigation. 

## 14. NT-3 

The biological effects of NT-3, the third member of the neurotrophin family, are produced via signalling by its high affinity receptor, TrkC, expressed predominantly on large diameter myelinated Aβ fibers in the DRGs and spinal cord, as well as on C-fibres [65]. NT-3 promotes neuronal growth and survival of sensory and sympathetic neurons in a manner similar to NGF [155,156]. NT-3 may also influence TrkA-positive neurons [157,158], but a functional role for NT-3-TrkA signalling remains to be elucidated. 

## 15. Pronociceptive Effects of NT-3 in Rodent Models

Initially, NT-3-TrkC signalling was thought to be confined to the modulation of proprioception [159]. However, anterograde transport of NT-3 from the lumbar DRGs into the spinal cord suggested a role in the modulation of nociceptive signalling in the spinal dorsal horn [61,65], but this remains to be investigated.

Intrathecally administered NT-3 at low doses (200 ng/day) for 20-days produced pronounced mechanical allodynia in the hindpaws of non-injured rats at 10–20 days after dose initiation [64]. The long latency for development of mechanical allodynia suggested that it is underpinned by complex central mechanisms that are unclear [64]. In other work, delivery of NT-3 (50 µg over 7 days) by osmotic minipump into the L5 DRGs of uninjured rats evoked temporary mechanical hypersensitivity that was significant at 1 but not 3 or 7-days after dose initiation [33]. 

## 16. Antinociceptive Effects of NT-3 in Rodent Models

NT-3 has been shown to inhibit the pronociceptive effects of NGF [65]. However, exogenous NGF had no effect on NT-3 expression levels in rodent DRGs, in contrast to the regulatory effects of NGF on BDNF expression [160]. Acute i.p. injection of NT-3 (10 or 20 mg/kg) evoked mechanical but not thermal hypoalgesia in the rat hindpaws that was underpinned by inhibition of SP release in the spinal cord that persisted in the presence of pharmacological concentrations of NGF [68]. Observations that naloxone reversed NT-3 induced inhibition of SP release in isolated rat spinal cords, suggested that cross-talk between NT-3 and inhibitory opioidergic signaling, may underpin NT-3 evoked antinociception [68]. Interestingly, other work has suggested that NT-3 may non-competitively block the binding of NGF to its high affinity receptor, TrkA thereby inhibiting NGF-mediated pronociceptive activity [157]. Alternatively, NT-3 may downregulate TrkA but not TrkC [158]. Irrespective of the mechanism, the onset of NT-3 antinociception is slow suggesting complexity [68].

## 17. NT-3 in Peripheral Neuropathic Pain

### 17.1. NT-3 in Rodent Models of Peripheral Nerve Ligation 

In CCI-rats, i.t. infusion of NT-3 for 7-days (600 ng/μL/h) both prevented and attenuated thermal, but not mechanical hyperalgesia in the ipsilateral (injured side) hindpaws [65]. This pain-relief was highly correlated with a marked decrease in the otherwise upregulated expression levels of TRPV1, phosphorylated (activated) p38 MAPK (pp38 MAPK) as well as voltage-gated sodium channels (Na_v_1.8 and Na_v_1.9) in the ipsilateral lumbar DRGs of these neuropathic rats [65,66]. Since these pronociceptive mediators were mainly driven by NGF, it is plausible to suggest that NT-3 is a powerful negative regulator of NGF and its pronociceptive activity [65].

### 17.2. NT-3 in Rodent Models of PDN 

In the lumbar DRGs of STZ-induced diabetic rats, suboptimal NT-3 dependent neurotrophic support and diabetes-induced deficits in axonal transport of NT-3 contributed to large fibre neuropathy [60]. Reversal of NT-3 depletion by intramuscular injection of adenovirus-based NT-3 gene therapy in STZ-diabetic rats, prevented development of diabetic neuropathy for up to 5-weeks diabetes induction [161]. In rats with impaired peripheral nerve conduction velocity at 8-weeks post-STZ administration, treatment with NT-3 for one month progressively reversed the deficit and normalised peripheral nerve conduction velocities to match those of non-diabetic rats [162]. 

### 17.3. NT-3 in Rodent Model of CIPN

In a rat model of cisplatin-induced CIPN, s.c. treatment with NT-3 reversed impaired sensory nerve conduction velocity, corrected abnormal neurofilament protein (NF200) distribution in large DRG sensory neurons, and reversed the large reduction in myelinated nerve fibre density in the sural nerves [59]. Importantly, sensory nerve conduction velocity was not altered in control rats administered the same NT-3 dosing regimen [59]. 

## 18. NT-3: Role in Central Neuropathic Pain 

### 18.1. NT-3 in Spinal Cord Injury Induced Neuropathic Pain Rodent Models 

Treatment of rats with complete spinal cord transection at T10 with an AAV-BDNF construct at the lesion site induced thermal hyperalgesia in the hindpaws as well as spasticity [163]. By contrast, AAV-NT-3 delivery to the site of spinal cord transection did not alter nociceptive thresholds in response to applied noxious thermal (heat) stimuli to the hindpaws [163]. 

### 18.2. NT-3 in Multiple Sclerosis-Associated Neuropathic Pain Mouse Model

The pathobiological role of NT-3 in rodent models of MS-induced neuropathic pain has not been investigated. However, as clinical data showed that NT-3 concentrations in peripheral blood mononuclear cells (PBMCs) were correlated strongly with widely accepted measures of brain atrophy in patients with MS, the NT-3 concentration in PBMCs may potentially exert a neuroprotective role in MS, but this requires future investigation [164]. 

## 19. NT-4 (NT-4/5)

Neurotrophin-4 was first isolated from *Xenopus* ovary and from snake venom [165]. Subsequently, it was found in mammals and reported as NT-4 [166] or NT-5 [167] and so it is often referred to as NT-4/5. NT-4, like BDNF, is a ligand for the TrkB receptor that is also synthesised in the DRGs and expressed in the spinal cord [55]. However, the biological effects of NT-4 differ from those of BDNF [168]. As NT-4 is expressed predominantly by motor neurons in the ventral horn of the spinal cord [56,169], this highlights NT-4’s key role in the maintenance and survival of motor neurons [56,170]. This notion is further supported by a study showing that NT-4 does not have a role in nociceptive transmission in *ex vivo* spinal cord preparations from mice null for NT-4, such that there were no deficits in the ventral root potentials evoked by stimulating nociceptive primary afferents [56].

## 20. NT-4: Lack of a Role in Neuropathic Pain 

Although there was a transient decrease in spinal cord levels of NT-4 in rats at 6 h post-sciatic nerve transection, NT-4 levels were normalised by 12 h [53]. Importantly, repeated i.t. injection of anti-NT-4 did not reverse thermal hyperalgesia in a mouse model of peripheral neuropathic pain, in contrast to the pain relief evoked by repeated i.t. administration of anti-BDNF [55]. These findings collectively affirm the notion that NT-4-TrkB signalling does not have a role in the modulation of nociception. Although i.pl. injection of NT-4 (200 ng) evoked transient thermal hyperalgesia in rats, it was reversed by 24 h [36]. Thermal nociceptive responses did not differ between NT-4 knockout and wild-type mice [171]. However, as morphine-induced analgesia was reduced in the knockout mice, NT4-TrkB signaling is implicated in opioid analgesia [171]. In other work, NT-4 expression levels were significantly reduced in the sciatic nerves of a rat model of PDN [57] and in the brain of EAE-mice [58]. Hence, the exact role of NT-4 in modulating nociceptive signaling remains to be defined. 

## 21. Proneurotrophins, p75NTR and Sortilin: Potential Modulatory Role in Pain

### 21.1. Proneurotrophins

Pro-NTs, like mature NTs, may be released extracellularly to exert their distinct biological actions not only via activation of p75NTR [4], but also via direct activation of Trk receptors [10,172] and indirectly after endocytosis and enzymatic cleavage [173]. To date, there is no direct evidence of a pronociceptive role for pro-neurotrophins [7] and so this knowledge gap requires future investigation. Nevertheless, observations that pro-NGF is upregulated in structures in close proximity to sprouting sensory and sympathetic nerve fibres, suggest a possible role in the pathogenesis of peripheral neuropathic pain [174]. Likewise, in our recent work that used our optimised mouse model of MS-induced neuropathic pain, neuropathic pain behaviour was significantly correlated with upregulated pro-BDNF expression and decreased mature-BDNF expression in the lumbar spinal cord [47]. As pERK expression was also increased in the lumbar spinal dorsal horn of these mice, our findings together suggests a pronociceptive role for pro-BDNF-TrkB-ERK signalling in MS-associated CNP [47]. In agreement, others have shown that pro-BDNF may exert TrkB-mediated downstream signaling, particularly ERK activation [10] that is implicated in central sensitisation and neuropathic pain development [175]. Clearly, future investigations to affirm (or not) that pro-NTs have direct pronociceptive effects by using mice null for p75NTR and/or i.t. treatment with recombinant pro-NTs or anti-p75NTR, are warranted. 

### 21.2. P75NTR

The p75NTR has diverse neuronal functions ranging from apoptosis to neuronal survival [176]. Apart from its traditionally known role in apoptosis, Schwann cell expression of the p75NTR suggested a possible role in remyelination after peripheral nerve injury [177]. In ~79% of all DRG neurons, co-expression of full length Trk receptor mRNA with p75 mRNA suggested involvement of the latter in transduction of the biological effects of NTs [178]. Pronociceptive signalling after L5 spinal nerve ligation in rats [179] induced neuropathic pain behaviour that was alleviated by pharmacological blockade of the p75NTR [179]. Upregulated expression of p75NTR, but not TrkA, in adjacent (L4) non-injured primary afferents was inhibited by anti-NGF [179]. Furthermore, mice null for p75NTR had a dramatic insensitivity to noxious thermal stimuli that was associated with decreased sensory nerve fibre innervations in the skin [180].

### 21.3. Sortilin

Sortilin, a type-1 membrane protein that is a member of the Vps10p-domain family of sorting receptors, is expressed widely in the PNS and the CNS [8]. A role for sortilin participation in NT signalling via complex formation with Trk receptors and/or p75NTR has been proposed [8,181]. It is well established that pro-NTs interact with the p75NTR-sortlin complex to mediate apoptosis [7]. Conversely, co-expression of sortilin with the p75NTR in sensory neurons unrelated to apoptosis, has been suggested to promote a range of biological activities including pronociceptive processing, but this remains largely unexplored [7]. 

## 22. Neurotrophin Induced Activation of Trk or p75 Receptors: Downstream Signalling

The NTs, particularly NGF and BDNF, signalling predominantly via their cognate high affinity receptors, TrkA and TrkB respectively, are implicated in the pathobiology of sensory neuron hyperexcitability and neuropathic pain [80]. Following NT-Trk receptor binding, dimerisation and autophosphorylation induced receptor activation and initiation of intracellular downstream signalling via multiple pathways [182]. These included Ras-mitogen-activated protein kinases (Ras-MAPK), phosphatidylinositol 3-kinase (PI3K) and phospholipase C-gamma-1 (PLC-γ1) [182]. Ras-MAPK activation promoted neuronal differentiation, neurite outgrowth and neuroplasticity via post-translational and transcriptional modifications [182,183]. Following NGF and BDNF induced activation of TrkA and TrkB respectively; augmented p38 MAPK and ERK signalling in the lumbar DRGs and spinal cord have been implicated in the pathobiology of chronic pain [127,184]. In other work, signalling via the Ras-MAPK pathway indirectly activated PI3K signalling [182], that in turn upregulated Akt signalling [185]. Activation of PLC-γ1, induced production of inositol tris-phosphate (IP3) and diacylglycerol (DAG) [186]. IP3 mobilised Ca^2+^ release from intracellular stores and activated Ca^2+^-regulated protein kinase C (PKC) as well as calmodulin-regulated protein kinase pathways [186]. By contrast, DAG production led to activation of DAG-regulated PKC isoforms [186]. Both IP3- and DAG-regulated signalling pathways were associated with synaptic and structural neuroplasticity [187] and PKC was implicated in the development of central sensitisation and persistent pain [143,188]. Despite the widespread expression of the p75NTR by sensory neurons in the DRGs and spinal cord, investigation of the contribution of NT-p75NTR to the pathobiology of neuropathic pain is in its relative infancy [80]. Generally, NT-p75NTR signalling is thought to primarily upregulate ceramide production and induce signalling via the Jun kinase pathway and induce NF-kappa B (NFκB) activation [189]. 

## 23. Clinical Studies Using rNTs or Anti-NTs for Pain Relief

Two decades ago, research in rodent neuropathic pain models suggested that exogenous NGF may alleviate PDN and CIPN [147]. However, in a randomised controlled clinical trial in patients with PDN, recombinant human NGF (rhNGF) produced pain/hyperalgesia at the injection site so severe that 22 patients out of 504 discontinued treatment [190]. The intolerable pronociceptive side-effects of rNGF hampered its testing in patients with CIPN [191]. 

Conversely, a randomised, double-blind, placebo-controlled clinical trial of tanezumab, a monoclonal antibody to NGF, showed that it produced significant analgesia in patients with osteoarthritis of the knee [11,192]. However, in a significant patient sub-group, osteoarthritis worsened such that some patients had to have total joint replacement [11,192]. Other frequent tanezumab-related adverse effects were arthralgia, pain in the extremities, paresthesia and peripheral edema [11,192]. Due to safety concerns, particularly the requirement for joint replacements in some patients, the United States Food and Drug Administration (FDA) placed all ongoing clinical trials of tanezumab for the relief of pain in patients with PDN [193], postherpetic neuralgia (PHN) [193] and low back pain [194] on clinical hold. However, very recently, the FDA has lifted this clinical hold [195,196,197]. An anti-TrkA monoclonal antibody, GBR900, is also in clinical trials as a potential novel analgesic that may have a superior safety profile compared with anti-NGF antibodies [198]. 

Clinical studies of anti-BDNF antibodies for the relief of chronic inflammatory and neuropathic pain conditions are lacking. This may be due to the unfavourable side effects that emerged during the early phase clinical trials of anti-NGF. Although the safety of NT-3 was established in healthy subjects [199], a Phase-1 clinical trial of NT-3 for relief of DPN and CIPN was discontinued in 1997 but no reports were published [200]. Preclinical research to date indicates that NT-4 does not have a role in the modulation of nociception and this may explain the lack of clinical trials of NT-4 for relief of neuropathic pain. 

## 24. Conclusions 

Considerable research to date has implicated a pathobiologic role for NGF and BDNF in neuropathic pain, whereas NT-3 generally appeared to alleviate neuropathic pain in rodent models. The conventional view is that the effects of NGF, BDNF and NT-3 in neuropathic pain are evoked by signalling via their cognate high affinity receptors, viz TrkA, TrkB and TrkC respectively. However, NGF, BDNF and NT-3 signalling via the p75NTR may also modulate neuropathic pain by either direct or indirect mechanisms, but additional research is needed. A role for pro-NTs signalling via their high affinity receptor, the p75NTR, and/or via low affinity binding to TrkA, TrkB or TrkC to modulate pro-nociceptive signalling in neuropathic pain, has also been suggested. However, research on this topic is in its relative infancy. 

Clinical research two decades ago showed that systemic administration of NGF evoked rather than relieved pain. This key observation re-directed efforts to the development of antibodies and small molecules that block rather than augment NGF-TrkA signalling, as a strategy to alleviate chronic inflammatory and neuropathic pain. Currently, multiple NGF antibody clinical trials in patients with a range of peripheral neuropathic pain conditions have recommenced following the decision of the US FDA in March 2015 to lift the clinical hold. 

Apart from clinical trial investigation of NGF antibodies as novel analgesics for relief of neuropathic pain, small molecule TrkA inhibitors are also in development as they may have a reduced risk for serious adverse-effects due to selective blockade of NGF-TrkA signalling whilst not impeding NGF-p75NTR signalling. However this remains to be determined in future investigations. 
